# Adjunctive Use of Circulating Tumor DNA Testing in Detecting Pancreas Cancer Recurrence

**DOI:** 10.3389/fonc.2019.00046

**Published:** 2019-02-06

**Authors:** Aixa E. Soyano, Candice Baldeo, Pashtoon M. Kasi

**Affiliations:** Department of Hematology and Oncology, Mayo Clinic, Jacksonville, FL, United States

**Keywords:** pancreas cancer, circulating tumor DNA (ctDNA), circulating tumor cells (CTC), liquid biopsy, relapse/recurrence

## Abstract

Liquid biopsies (circulating tumor DNA—ctDNA testing) are increasingly being utilized in clinical trials as well as practice for the detection of cancer, monitoring of tumor genomic abnormalities, response to treatment and early detection of relapse/recurrence. Here, we present a challenging case where liquid biopsy was used to confirm an early recurrence of pancreatic cancer where acquisition of tissue was not safe or feasible on more than one occasion.

## Background

In cancer, surgical or interventional biopsies are obtained traditionally to characterize the site of origin of the cancer cells as well as to potentially characterize the genetic profile of the tumor. These approaches only represent a limited snap shot of the tumor (1). Furthermore, it is known that cancers can evolve on treatment and are known to have intratumoral and intertumoral heterogeneity. Single tumor biopsies can limit the extent of personalized medicine as they can underestimate the tumor genomic landscape and evolution throughout treatment (2).

Liquid biopsies have been developed recently and improved over time as a potential surrogates for tumor biopsies in cancer screening, detection of genomic alterations, determination of response to treatment, and detection of early recurrence (3). A lot of research is still underway. At present their use is primarily limited to advanced/metastatic cases in practice.

We present a case of a woman with pancreatic cancer were a liquid biopsy was used twice for confirmation of recurrence and prompt initiation of treatment in lieu of a surgical biopsy due to the difficulty of obtaining tissue to confirm recurrence. Serial evaluations by liquid biopsy confirmed response to treatment and then later again recurrence. In all these instances, it was not safe or feasible to obtain tissue.

## Introduction (Case)

A 70 years old Caucasian female with a history of laparoscopic Roux-en-Y gastric bypass in 2013 complicated by the development of a large ventral hernia in May 2014 was evaluated. As part of a pre-surgical evaluation a CT of the abdomen on December 8, 2015 identified a 3.5 × 2.8 cm mass in the head of the pancreas. MRI of the abdomen on December 29, 2015 showed the mass to be 3 × 2.9 × 3.4 cm. CT chest showed no pulmonary metastases. On January 11, 2016, she underwent open pylorus-preserving pancreaticoduodenectomy, cholecystectomy, and repair of the ventral hernia. Pathology showed a 3.5 cm, invasive well differentiated pancreatic ductal adenocarcinoma, arising in a background of intraductal papillary mucinous neoplasm (IPMN). The tumor invaded the duodenal wall, peripancreatic soft tissues, and extrapancreatic common bile duct. Margins were negative for tumor. IPMN was present at the pancreatic surgical margin without evidence of high-grade dysplasia/carcinoma. Fourteen of twenty-seven regional lymph nodes were positive for metastatic carcinoma (14/27). Lymphovascular invasion was indeterminate. Perineural invasion was present. Final pathologic staging per TNM classification was IIB (pT3pN1M0). Comprehensive tumor based genetic testing showed mutations in KRAS G12V, CDKN2A p16INK4a A17fs^*^21, TP53 S149fs^*^32, and *U2AF1 S34F*.

She initiated adjuvant therapy with gemcitabine on February 2016 and completed 2 cycles through April. Course was complicated by abdominal pain and rash. CT abdomen/pelvis with contrast on April 11, 2016 showed interval appearance of a solid mass in the tail of the pancreas worrisome for a new primary cancer. PET scan showed the mass to be hypermetabolic with an SUV 6.4. MRI showed postop Whipple procedure with new hypoenhancing mass in the tail of the pancreatic remnant measuring 1.8 × 2.1 cm, correlating with the hypermetabolic lesion seen on PET scan. There was no evidence of liver metastases. CA 19–9 tumor marker was 12 U/mL (normal <55 U/mL).

On May 10, 2016, she underwent splenectomy, remnant gastrectomy, and total pancreatectomy. Operative note did not report any visible abdominal malignancy. Pathology showed IPMN with focal high-grade dysplasia but no overt cancer. Seven peripancreatic and 4 peri hilar (splenic) nodes were negative for malignancy. Immunohistochemistry showed no expression for PD-L1 and normal expression of *MLH1, PMS2, MSH2*, and *MSH6*. Postoperative course had a slow recovery. She declined resumption of adjuvant therapy (either radiation or chemotherapy) and she was surveilled with imaging.

On May 12, 2017 a CT of the chest, abdomen and pelvis showed a newly enlarged 1.2 cm low para-aortic lymph node, suspicious for metastatic disease. A single 1.1 cm periportal lymph node was also mildly increased in size from prior. Her case was discussed in the multidisciplinary tumor board. The para-aortic node was in a challenging location for successful biopsy. CA 19-9 was 34 U/mL (normal <55 U/mL). A circulating tumor DNA test (ctDNA, Guardant360®) was sent on May 22, 2017 that showed mutations in *CDKN2A*—that was present at baseline tumor based genetic profile and a new mutation in *ARID1A* (T2138del).

After a thorough discussion with the patient the decision was made to start systemic chemotherapy treatment with Gemcitabine/nab-paclitaxel for recurrent adenocarcinoma of the pancreas. She completed 4 cycles of Gemcitabine/nab-paclitaxel. This was followed by chemoradiation with capecitabine as a radiosensitizer, which she completed in January 2018. A repeat ctDNA in February 2018 showed disappearance of the previous *CDKN2A* and *ARID1A* mutations and no new mutations were detected.

Unfortunately, in March 2018 imaging again showed recurrence in the lungs and liver. The locations still were not amenable to a tissue biopsy and liquid biopsy was utilized that picked up again mutations that were concordant with the patient's tumor (Table 1). She is currently on chemotherapy with liposomal irinotecan/5-fluorouracil.

**Table 1:** Comparison of aberrations detected on baseline tumor tissue based comprehensive genetic testing and later in circulating tumor DNA (ctDNA) at 3 distinct timepoints.

**Baseline comprehensive tumor based testing**	**ctDNA timepoint 1 (recurrence #1)**	**ctDNA timepoint 2 (post-chemoradiation)**	**ctDNA timepoint 3 (recurrence #2)**
	0.09% Highest variant allele fraction	ND	0.2% highest variant allele fraction
***KRAS G12V***	ND	ND	***KRAS G12V***
CDKN2A p16INK4a A17fs*21	CDKN2A p16INK4a A17fs*21	ND	ND
TP53 S149fs*32	ND	ND	TP53 S149fs*32
***U2AF1 S34F***	ND	ND	ND
***VUS***			
*ARID1A R1869Q*	ND	ND	ND
FOXP1 *115Lext*?	ND	ND	ND
*PIK3R2 P261_S262insP*	ND	ND	ND
*PLCG2 Q387P*	ND	ND	ND
ND	*ARID1A T2138del*	ND	ND
ND	ND	ND	*IDH2 G145G*

*Chronological results of circulating tumor DNA (Guardant 360) showing mutations at baseline, at the time of recurrences and post treatment. ND, not detected; ctDNA, circulating tumor DNA; VUS, variant of unknown significance. In bold are the deleterious mutations noted as opposed to variants of unknown significance*.

Written informed consent was obtained from the patient for the publication of this case report.

## Guardant 360

As per the manufacturer, the “Guardant 360 is a whole blood based cell free DNA detection assay. A Guardant sample collection kit is used to obtain two 10 mL of whole blood from the patient. The sample is sent directly to the laboratory at Guardant Health. The test detects single nucleotide variants in a targeted panel of 73 genes, and selected copy number amplifications, fusions/rearrangements, and indels for a specific set of genes. All four types of genomic alterations are reported in a single test. Turnaround time for testing is approximately ≤ 14 days.

The genes sequenced include: *AKT1; ALK; APC; AR; ARAF; ARID1A; ATM; BRAF; BRCA1; BRCA2; CCND1; CCND2; CCNE1; CDH1; CDK4; CDK6; CDKN2A; CTNNB1; DDR2; EGFR; ERBB2; ESR1; EZH2; FBXW7; FGFR1; FGFR2; FGFR3; GATA3; GNA11; GNAQ; GNAS; HNF1A; HRAS; IDH1; IDH2; JAK2; JAK3; KIT; KRAS; MAP2K1; MAP2K2; MAPK1; MAPK3; MET; MLH1; MPL; MTOR; MYC; NF1; NFE2L2; NOTCH1; NPM1; NRAS; NTRK1; NTRK3; PDGFRA; PIK3CA; PTEN; PTPN11; RAF1; RB1; RET; RHEB; RHOA; RIT1; ROS1; SMAD4; SMO; STK11; TERT; TP53; TSC1;* and *VHL*. Covered exons are completely sequenced to maximize detection of known somatic variants. Sensitivity for genes sequenced is >99.9% if the allelic fraction/copy number is >0.25% with a positive predictive value (PPV) of 99.6. If the allelic fraction is 0.05–0.25% the sensitivity of the test is 63.8% with a PPV of 92.1%.

The following genes are also analyzed for copy number amplifications (CAN): *AR; BRAF; CCND1; CCND2; CCNE1; CDK4; CDK6; EGFR; ERBB2; FGFR1; FGFR2; KIT; KRAS; MET; MYC; PDGFRA; PIK3CA*; and *RAF1*. Sensitivity for CAN is 95% and PPV is 100%.

Genes analyzed for fusions/rearrangements are: *ALK; FGFR2; FGFR3; NTRK1; RET*; and *ROS1*. Sensitivity and PPV for fusions is 100% if allelic fraction is ≥0.3%.

The following genes are also analyzed for indels: *APC; ARID1A; ATM; BRCA1; BRCA2; CDH1; CDKN2A; EGFR; ERBB2; GATA3; KIT; MET; MLH1; MTOR; NF1; PDGFRA; PTEN; RB1; SMAD4; STK11; TP53; TSC1;* and *VHL*. Sensitivity for indels is >99.9% and PPV is 98% if allelic fraction >0.25%.”

## Discussion

Pancreas cancer represents the 4th leading cause of cancer deaths in both men and women in the United States. In contrast to the improved survival seen in multiple cancer types the progress in improvement in overall survival has been slow for pancreatic cancer with an overall 5 year survival rates of approximately 8% (4). This could be in part secondary to the majority of patients presenting with advanced disease at diagnosis. New biomarkers for diagnosis and monitoring treatment of this disease are required to help improve outcomes.

Cancer cells can release cell fragments and dead cells into the circulation. Liquid biopsies rely on analysis of tumor material such as DNA (known as circulating tumor DNA or ctDNA), RNA, proteins, exosomes and/or whole cells (known as circulating tumor cells or CTCs) that can be found in blood, cerebrospinal fluid, saliva, or urine. They have been developed with the goal of detecting the material in a sample that originates from cancer cells (5). They can be detected by several techniques including quantitative real time polymerase chain reaction (PCR), methylation specific or digital PCR, next generation sequencing and/or BEAMing (beads, emulsion, amplification, and magnetics) (6).

A potential application of liquid biopsies is detecting cancer at an early stage when treatment may be most successful; however a concern is a false positive results and/or overtreatment of tumors that may be more harmful than the tumor itself. Another potential application of liquid biopsies is the paradigm of precision medicine by identification of unique molecular characteristics of a tumor that could be used to determine the optimal treatment. Most importantly, it also allows for simultaneous testing of multiple genes depending on the platform used that can be specific for certain types of cancer.

Liquid biopsies can also be used as prognostic or predictive markers. For example, a prospective study by Toledo et al. of 25 patients with newly diagnosed wild type RAS metastatic colorectal cancer treated with FOLFIRI-cetuximab used liquid biopsies by BEAMing for validation and monitoring of ctDNA mutations in *KRAS, NRAS, BRAF*, and *PIK3CA*. They found that patients with prolonged responses to treatment with anti EGFR therapy maintained a wild type *RAS* status. In contrast, patients who showed upsurges in circulating *KRAS* mutations had rapid disease progression with clinical deterioration and spread of metastasis (7).

A widely studied application for liquid biopsies is monitoring treatment response and predicting early relapse/recurrence. Namlos et al. reported a case of a patient with high grade soft tissue sarcoma were they prospectively collected primary tumor sample at diagnosis and several plasma (ctDNA) samples during the disease course. Targeted resequencing of the levels of ctDNA allowed them to detect progression of the disease 6 weeks after surgery and this was corroborated by detection of multiple new metastatic sites on imaging (8). Nakamura et al. retrospectively reviewed ctDNA in 17 patients with several hematological malignancies who achieved remission after first line chemotherapy. Eight patients in the relapsed group had more than doubled the levels of ctDNA at several time points and there was a median 30-days lead-time over clinical relapse. In contrast, in the 9 patients from the remission group, ctDNA remained undetectable (9). Another example from a prospective study by Birkemkamp-Demtröder et al. in 60 advanced bladder cancer patients used ctDNA in plasma and urine to detect metastatic relapse after cystectomy and measure treatment efficacy. Patients with metastatic relapse had higher ctDNA levels compared to disease free patients (*P* < 0.001) and the median positive lead-time between ctDNA detection in plasma and diagnosis of relapse was 101 days (range 0–932). A meta-analysis of the use of liquid biopsy (both CTCs and ctDNA) as a predictor of recurrence after surgery of non-small cell lung cancer showed that positive blood CTCs or ctDNA after surgery was significantly associated with worse progression free survival [Hazard ratio (HR) 3.37, 95% CI 2.28–4.96, *p* < 0.001 and HR 8.15, CI 2.11–31.50, *p* = 0.002, respectively]. One and two year's recurrence rate were higher in both the CTCs and ctDNA groups compared to the negative groups (10).

In pancreatic cancer specifically, few studies have been reported in this area. Sausen et al. demonstrated that the presence of CTCs after resection of the primary tumor did predict relapse and worse outcomes with recurrence detected at a median of 6 months earlier than CT imaging (11). Furthermore, Ren et al. showed a presence of 80.5% of CTCs in 41 advanced pancreatic cancer patients at baseline prior to initiation of 5-fluorouracil based chemotherapy. After 1 week of treatment the presence of CTCs decreased to 29.3% suggesting a potential role for using CTCs as a biomarker for treatment response in this malignancy (12).

Future approaches would include using ctDNA alone or in combination with other imaging or laboratory tests as a marker for early detection of recurrence in pancreas cancer. Currently, an ongoing prospective clinical trial in Korea is evaluating the use of ctDNA for early screening of recurrence of pancreas cancer and its correlation with clinical outcomes (NCT 02934984), which will also help discuss optimal timing of liquid biopsies in monitoring for recurrence. Furthermore, Cohen et al. described that the combination of ctDNA with protein biomarkers (i.e., tumor markers) increased the sensitivity of detection of resectable pancreatic cancer with a sustained high specificity (13).

In the case of our patient the rationale behind using a liquid biopsy to help detection or confirmation of pancreas cancer recurrence was the inability to obtain tissue twice due to the difficult and inaccessible location of the suspected recurrence. Even though ctDNA is not necessarily meant for that purpose, the results which were taken into considerations with patient's underlying prior comprehensive tissue based tumor testing were considered sufficient alongside the clinical and radiographic picture. The positive result of the ctDNA, the inability to obtain a tissue biopsy and a slight worsening of the overall clinical condition represented red flags that recurrence/relapse was underway and treatment needed to be initiated soon to help achieve the best clinical outcomes.

It is interesting to note that in our case the *KRAS* and *TP53* mutations were not detected after the initial chemotherapy regimen. While clones/subclones can evolve, given this is pancreas cancer and the mutations that were not detected were *KRAS/TP53*, it is likely that in those instances cell free DNA was below 0.25% allelic fraction/copy number and was not detected by the test. So it was falsely negative since tumor burden can impact the sensitivity of the assay whereby a positive test is helpful but a negative test could be negative as a consequence of limited tumor burden. It is also important to realize that clonal hematopoiesis of indeterminant potential (CHIP) is an entity that can be misinterpreted as ctDNA. Comparison of test results with baseline tissue based tumor testing and interpreting results in context of the particular tumor can help.

To our knowledge this is the first case reported of realtime clinical use of liquid biopsy to confirm recurrence twice in a patient with pancreas cancer when tissue biopsy was not considered safe or feasible. Liquid biopsies are safe, tolerable, and sensitive tools that can be incorporated into routine clinical care of cancer patients to help with detection of early recurrence/relapse. It is important to have baseline comprehensive tumor based genetic testing to avoid erroneous diagnoses from clonal hematopoiesis of indeterminate significance. As noted in Table 1, given different coverage, levels of ctDNA, and testing platforms, results of these assays may not always be concordant. This further argues to not to interpret such results in the absence of baseline tumor based genetic testing.

## Concluding Remarks

Our article highlights a real-time example of using a commercially available assay to help with confirmation of a clinically suspicious recurrence event in a patient with inaccessible lesions that were not deemed safe for a biopsy. We cannot make any conclusions about if we made an impact on the patient's overall survival by acting early on recurrence. A larger study would be needed to formally study this. However, in the right patient's context with baseline tumor based genetic testing results available, ctDNA testing can be of considerable value.

## Author Contributions

All authors listed have made a substantial, direct and intellectual contribution to the work, and approved it for publication.

### Conflict of Interest Statement

The authors declare that the research was conducted in the absence of any commercial or financial relationships that could be construed as a potential conflict of interest.

## Abbreviations

BEAMing: beads, emulsion, amplification, and magnetics
CT: computed tomography
CTC: circulating tumor cells
cfDNA: circulating free DNA
ctDNA: circulating tumor DNA
DNA: deoxyribonucleic acid
IPMN: intraductal papillary mucinous neoplasm
PCR: polymerase chain reaction
PET: positron emission tomography
RNA: ribonucleic acid.
